# Research on Audio Recognition Based on the Deep Neural Network in Music Teaching

**DOI:** 10.1155/2022/7055624

**Published:** 2022-05-27

**Authors:** Yun Cui, Fu Wang

**Affiliations:** ^1^School of Music and Performing Arts, Mianyang Teachers' College, Mianyang 621000, China; ^2^College of Management Science, Chengdu University of Technology, Chengdu 610059, China

## Abstract

Solfeggio is an important basic course for music majors, and audio recognition training is one of the important links. With the improvement of computer performance, audio recognition has been widely used in smart wearable devices. In recent years, the development of deep learning has accelerated the research process of audio recognition. However, there is a lot of sound interference in music teaching environment, which leads to the performance of the audio classifier that cannot meet the actual demand. In order to solve this problem, an improved audio recognition system based on YOLO-v4 is proposed, which mainly improves the network structure. First, Mel frequency cepstrum number is used to process the original audio and extract the corresponding features. Then, try to apply the YOLO-v4 model in the field of deep learning to the field of audio recognition and improve it by combining with the spatial pyramid pool module to strengthen the generalization ability of data in different audio formats. Second, the stacking method in ensemble learning is used to fuse the independent submodels of two different channels. Experimental results show that compared with other deep learning technologies, the improved YOLO-v4 model can improve the performance of audio recognition, and it has better performance in processing data of different audio formats, which shows better generalization ability.

## 1. Introduction

Music is an abstract art form with sound as its means of expression. In the process of music teaching, solfeggio can strengthen students' musical memory ability, enable students to accurately identify music works, and thus obtain better “musical perception.” As an important link in solfeggio, audio recognition training is very difficult for junior students. This is because students need to master all kinds of clefs, distinguish the length and duration represented by different notes, and the pitch difference between different notes.

Audio signal analysis based on embedded intelligent devices has attracted more and more researchers' attention [1–7]. Intelligent wearable devices with audio recognition function can help students solve the above problems and realize music teaching assistance. The task of audio recognition needs to preprocess the collected audio signals first, extract useful features for distinguishing music scores from them, and finally classify them according to these features. Classification is a very important method of data mining [8–10]. Classification refers to generating a classification function according to certain rules on the basis of training set data. This function can map the data of the test set to one of the given categories, thus realizing the category prediction of unknown data. At present, common classifiers include decision tree, logistic regression, support vector machine (SVM), Naive Bayes, *k*-nearest neighbor algorithm (KNN), BP neural network, and deep learning [11–13].

The previous machine learning methods often need to manually extract the features that can represent the original data as the input of the classifier. However, deep learning can automatically extract the high-dimensional features of samples (without manual feature extraction), as long as the input data cover the information of the original data as much as possible, which is suitable for large-scale data. The deep learning method can realize specific audio recognition tasks with the help of a large amount of audio data collected by intelligent devices. The convolutional neural network (CNN), as a kind of deep learning architecture, is widely used in image classification, speech recognition, natural language processing, and other fields because of its superior performance in local feature learning [14]. Different from other neural network models (such as Boltzmann machine and recurrent neural network), the CNN characterized in that core operation is convolution operation. The YOLO network draws lessons from the CNN classification network structure and shows good advantages in the field of image recognition, which has attracted the attention of many researchers.

Therefore, this study tries to apply the YOLO-v4 model to the field of audio recognition and improves its network structure. In addition, the stacking method in ensemble learning is used to fuse two independent submodels of different channels, and the classification performance of the fused system is further improved compared with the single submodel.

## 2. Related Works

Nowadays, with the emergence of a large number of smart devices, the excellent computer performance and the development of deep learning technology have jointly promoted the research process in the audio field. Combined with the main research contents of this study, the current research status will be introduced from two aspects: convolutional neural network and audio recognition.

The convolutional neural network structure originated from a study by Yann LeCun in 1998 is called the Le Net-5 artificial neural network. The convolutional neural network, like other neural networks, can be trained by the back propagation algorithm [15]. In 2012, Alex Krizhevsky and others adopted CNN technology for the first time in complex computer vision tasks. By using 3 fully connected layers, 5 convolution layers, and Softmax classifier, a convolutional neural network with 8 layers is constructed, which is named AlexNet. AlexNet uses ReLU activation function, and at the same time, it also uses regularization (dropout) to prevent overfitting. In 2014, the Google' computer vision team puts forward the GoogLeNet network [16], with a network depth of 22 layers, which contains a new structure, incident. It integrates the features of different depths and the same scale, and the detection accuracy is improved. On the basis of the GoogLeNet network, YOLO and SSD algorithms appeared. Both methods are based on a single end-to-end network, which can complete the input from the original image to the output of the object position and category.

In the aspect of audio recognition, Yang and Zhao [17] proposed an acoustic scene classification method based on the support vector machine (SVM), which enhanced the sound texture to improve the classification accuracy. Greco et al. [18] proposed a voice recognition system based on the heuristic deep learning method. Demir et al. [19] proposed a new pyramid cascade CNN method for environmental sound classification. Zhu et al. [20] proposed an improved YOLO-v4 algorithm for sound imaging instruments, which effectively improved the accuracy of acoustic phase cloud image detection. The above methods all show excellent performance in dealing with audio recognition tasks in a single acoustic scene, but there are many sound disturbances in the music teaching environment, and it is necessary to deal with a variety of different audio format data.

Therefore, this study proposes an audio recognition system based on the improved YOLO-v4 network model. The main innovations and contributions include the following: (1) try to apply YOLO-v4 network architecture, which is excellent in the field of deep learning, to the field of audio recognition, and improve it by combining the spatial pyramid pool module. The improved YOLO-v4 network architecture effectively utilizes the spatial information in audio files, thus strengthening the generalization ability of data in different audio formats. (2) The stacking method in ensemble learning is used to fuse two independent submodels of different channels, and the classification performance of the fused system is improved.

## 3. Extraction and Processing of Audio Features

Extracting the best parameter representation of audio signal is one of the important tasks to produce better recognition performance. The feature extraction in this stage is very important for the classifier classification in the next stage because it will directly affect the classification efficiency.

In the classification task, especially the audio classification task, the Mel frequency cepstrum coefficient (MFCC) which describes the spectral shape has a long history. Although the MFCC extraction process will cause lossy compression of data, its classification and recognition effect are quite available even when the data rate is very low. In addition, compared with other classification features, MFCC is widely used because it is more in line with the auditory frequency response curve of human ears.

**Figure 1:**
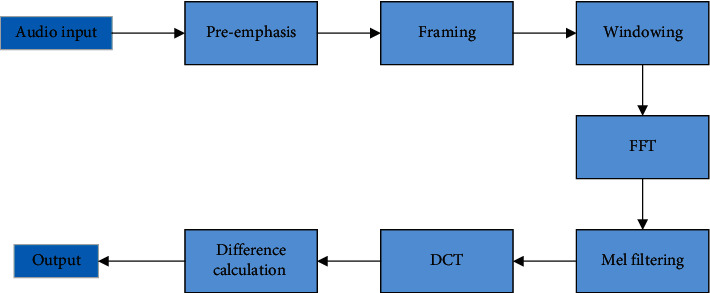
MFCC feature extraction steps.

The reason why human beings can judge different environments in complex sound environment lies in the credit of the cochlea. The cochlea can be seen as a filter bank to help people filter 20-20 kHz audio. The problem is that the sensitivity of the cochlea to frequencies in the auditory range is not linear, but there is a mapping relationship. MFCC can simulate the frequency response of the human ear. MFCC feature extraction consists of seven steps, and the whole process is shown in Figure 1.

Common audio signals have the phenomenon that the low-frequency energy is large, but the high-frequency energy is small. If it is transmitted directly, it will lead to high signal-to-noise ratio at low frequency and insufficient signal-to-noise ratio at high frequency. In order to make up for this loss of audio signal during transmission, preemphasis is introduced to compensate the input signal, so that the high-frequency characteristics of audio signal can be highlighted. Preemphasis is usually achieved by means of a high-pass filter [21–23].

Let the voice sample value at the *n*^th^ time be *X*[*n*], and the result after preemphasis is

(1) $$\begin{array}{c} Y\left[n\right]=X\left[n\right]-aX\left[n-1\right], \end{array}$$

where *a* is the preemphasis coefficient, usually within 0.9-1.0.

**Figure 2:**
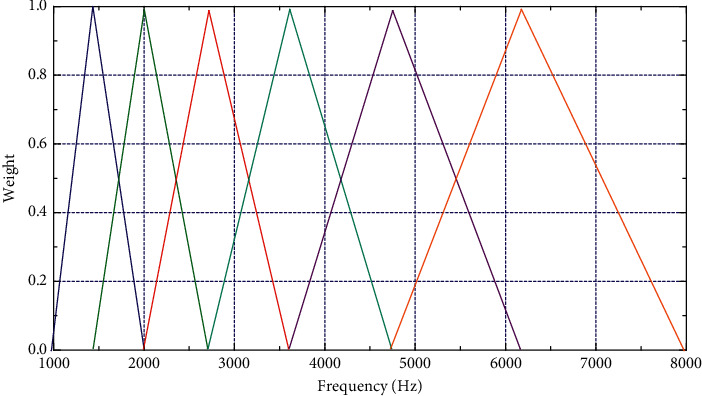
Mel scale filter bank.

Framing divides audio samples obtained from analog-to-digital conversion (ADC) into small frames with a length in the range of 20–40 milliseconds. After preemphasis and framing are completed, it is necessary to add a Hamming window to each frame. Windowing is to control the amount of data processing, and only the data in the window are processed at a time. The frequency range in the fast Fourier transform spectrum is very wide, which leads to the speech signal not following the linear scale [24–26]. Therefore, it is necessary to pass the Mel scale filter bank as shown in Figure 2.

Figure 2 shows a set of triangular filters, which are used to calculate the weighted sum of the spectral components of the filters, so that the processed output approximates Mel scale. The amplitude-frequency response of each filter is triangular. The Mel spectrum of a given frequency *f* is calculated as follows:

(2) $$\begin{array}{c} F\left(\text{Mel}\right)=2595\cdot {\text{log}}_{10}\left(1+\frac{f}{700}\right). \end{array}$$

Discrete cosine transform (DCT) transforms the Mel spectrum into time domain. The result of the transforms is called Mel frequency cepstrum coefficient. The coefficient set is called acoustic vector. Therefore, each input is converted into an audio vector sequence.

In order to improve the signal recognition performance, the differential spectrum based on the static characteristics of audio signals is used to describe the dynamic characteristics of audio signals. 13 first-order difference features and 39 second-order difference features are introduced. The frame energy of signal *x* in the window from time *t*_1_ to *t*_2_ is as follows:

(3) $$\begin{array}{c} \text{Energy}=\sum_{t=t_{1}}^{t_{2}}X^{2}\left(t\right). \end{array}$$

13 first-order differential features represent the changes between frames of cepstrum in MFCC features, while 39 second-order differential features represent the changes between frames in first-order differential features. The first-order difference is calculated as follows:

(4) $$\begin{array}{c} d\left(n\right)=\frac{c\left(n+1\right)-c\left(n-1\right)}{2}, \end{array}$$

where *c*(*n* + 1) represents the cepstrum coefficient at time *n* + 1.

## 4. SPP-YOLO-v4 Network Structure

### 4.1. Spatial Pyramid Pool (SPP) Module

**Figure 3:**
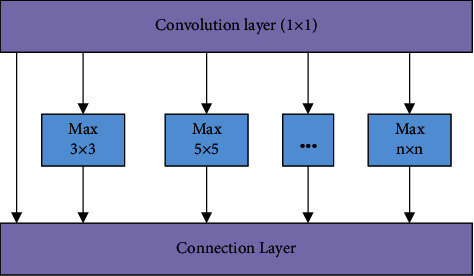
Basic module structure of SPP.

SPP can avoid information distortion caused by scaling, stretching, clipping, and other operations and provide output that is not affected by the input size, which cannot be achieved by sliding window pooling technology [27]. Second, SPP can pool with multiple scales, while sliding window pooling only uses one window scale. The basic structure of the SPP module is shown in Figure 3. It can be seen that because the input size is flexible, SPP can combine the features of data in different audio formats. The dimension of the transformed feature vector is the same as that of the fully connected layer, while alleviating the generalization problem.

### 4.2. SPP-YOLO-v4

**Figure 4:**
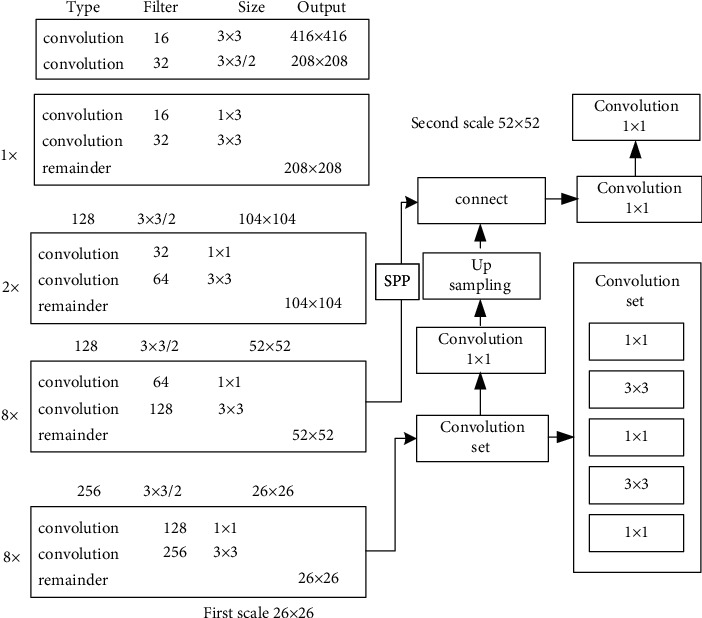
SPP-YOLO-v4 network structure.

YOLO-v4 is a high-precision real-time single-stage detection algorithm integrating YOLO-v1, YOLO-v2, and YOLO-v3. YOLO-v4 constructs the CSP cross-stage partial network (CSPNet) in the residual module, in which the feature layer is the input and the feature information of the higher layer is the output. This shows that the learning objectives of YOLO-v4 in the ResNet module are different between output and input. Therefore, residual learning is realized, and the model parameters are reduced, so the feature learning ability is enhanced. Considering the application environment of music teaching, some changes are made on the basis of the original network, and the final network structure is shown in Figure 4.

First, the feature layer is convolved three times, and then, the input feature layer is maximally pooled by using the maximum pooled cores of different sizes. After convolution and upsampling, different feature layers are connected in series to realize feature fusion. Then, perform downsampling, compress height and width, and finally stack with the previous feature layer to realize more feature fusion (5 times). The classification module uses the features extracted from the network to make classification judgment. Take the 13 × 13 grid as an example, which is equal to dividing the input Mel spectrogram into 13 × 13 squares; then, each square will be preset with three prior frames. The classification results of the network will adjust the positions of these three prior boxes and finally filter by the nonmaximum suppression (NMS) algorithm [28], so as to get the final classification results.

## 5. Audio Recognition System Based on SPP-YOLO-v4

### 5.1. System Architecture

**Figure 5:**
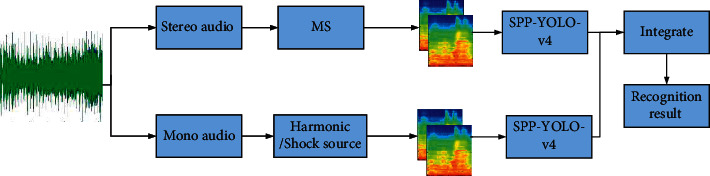
Overall architecture of the audio recognition system.

As shown in Figure 5, after audio input, the proposed audio recognition system first divides the audio sequence data into two parts. The first part comes from stereo channel, while the second part is compressed into mono. The audio signals of the two channels are extracted by MFCC spectrogram and input into the SPP-YOLO-v4 model as features. Then, two groups of SPP-YOLO-v4 models are integrated, and the stacking method is adopted in the integration. After the integrated learning of the two models, the audio classification results are finally output. The details of the SPP-YOLO-v4 model are shown in Figure 4.

### 5.2. Stacking Integrated Learning

As shown in Figure 5, the system uses ensemble learning technology to get the final classification result. The basic idea of ensemble learning is to form a strong classifier through the combination of several weak classifiers. Even if some weak classifiers make wrong predictions, they can be corrected by other weak classifiers with correct predictions, thus achieving the effect of improving the system performance.

Assuming that *x* is an input, *m*_*i*_ (*i*=1,2,…, *k*) is a group of classifiers and the output of the classifiers is the probability distribution *m*_*i*_(*x*, *c*_*j*_) of each class *c*_*j*_ (*i*=1,2,…, *k*), the final output *y*(*x*) of the integrated classifier can be expressed as

(5) $$\begin{array}{c} y\left(x\right)=\underset{c_{j}}{\text{argmax}}\sum_{i=1}^{k}w_{i}m_{i}\left(x,c_{j}\right), \end{array}$$

where *w*_*i*_ is the weight of classifier *m*_*i*_. Ensemble is a method to calculate the best weight of each classifier according to the classification target. At present, popular ensemble learning algorithms include stacking, bagging, boosting, ensemble selection, and so on. The ensemble learning algorithm selected in this study is the stacking method.

**Figure 6:**
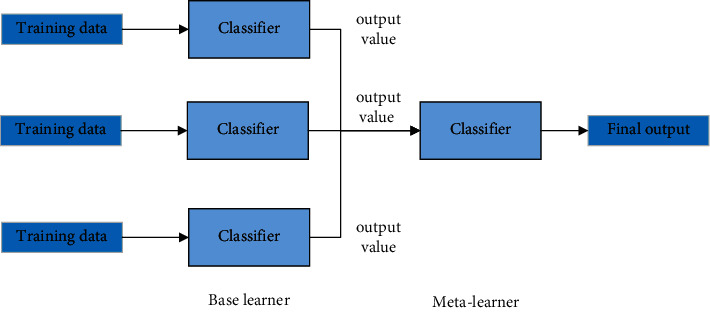
Basic principle of the stacking method.

Stacking is a process of second-order learning with the output of the first-order learning process as input, also known as “meta-learning.” The stacking method has become a popular ensemble learning method, not only because its implementation is quite simple but also because it can significantly improve the generalization ability of the system, which is very consistent with the purpose of this study. The basic principle of the stacking method is shown in Figure 6.

## 6. Experiment and Result Analysis

### 6.1. Experimental Environment and Dataset

The hardware platform of this study is Intel Core i3-M350 CPU@ Dual-core 2.20 GHz, 8 GB of DDR2 memory, Nvidia RTX2080Ti GPU, and 11 GB of video memory. The PyCharm integrated development tool is developed in Python 3.5.0 language. The YOLO annotation framework written in Python is used to convert the numerical format, so that it can be read by YOLO. The comparison methods are the Gaussian mixture model (GMM), CNN, and R-CNN.

The experimental dataset is recorded audio files in the real teaching environment. The dataset consists of audio types of four different labels (D1, D2, D3, and D4). All audio files are cut into 30-second clips. There are 12 audio file formats including MPEG, MP3, and WMA. Each recording is performed at a different location, and the average recording duration is 3–5 minutes. The recording equipment includes two-channel Soundman OKM II Classic/studio A3 in-ear microphone and Roland Edirol R09 waveform recorder with 44.1 kHz sampling rate and 24 bit resolution.

The used dataset contains 1404 audio files, and the number of audio files of each type is 351. About 70% of the data is used for training the audio recognition model, and the remaining 30% is used for testing. The system settings are given in Table 1.

### 6.2. Evaluation Criteria

The mean accuracy (mAP) is calculated as follows:

(6) $$\begin{array}{c} \text{mAP}=\int_{0}^{1}p\left(\tau \right)d\tau , \end{array}$$

where *p*(*τ*) is the accuracy of audio classification.

Precision and recall are defined as follows:

(7) $$\begin{array}{c} \mathrm{Pr}=\frac{\text{TP}}{\text{TP}+\text{FP}},\\ \text{Recall}=\frac{\text{TP}}{\text{TP}+\text{FN}}, \end{array}$$

where TP is the positive alarm rate, FP is the false alarm rate, and FN is the missed alarm rate.

*F*1 score is the harmonic value of precision and recall rate. The higher the value, the better the performance. It is defined as follows:

(8) $$\begin{array}{c} \text{F}1=2\frac{\text{Recall}\times \mathrm{Pr}}{\text{Recall}+\mathrm{Pr}}. \end{array}$$

### 6.3. Verification of SPP-YOLO-v4 Performance

In order to verify the promotion effect of the proposed improved YOLO-v4 (SPP-YOLO-v4) on generalization ability, it is compared with the traditional YOLO-v4 model. In the experiment, 3 of 12 audio file formats were selected: MPEG, MP3, and WMA. The generalization ability of SPP-YOLO-v4 is given in Table 2.

From Table 2, it can be found that the overall accuracy of SPP-YOLO-v4 is higher than that of traditional YOLO-v4, which verifies its generalization ability for data in different audio formats. This is because compared with the original method, SPP of SPP-YOLO-v4 contains more layers, but it also increases the processing time.

### 6.4. Comparison of Test Results

Table 3 provides the results of training loss, mAP, and so on for all categories after 8000 rounds of training. It can be seen that the training model of the proposed method can effectively identify audio types. It has certain advantages in accuracy, recall rate, and *F*1 score, and its loss value is also the lowest of all methods, only 0.0122. Therefore, the stability and accuracy of the proposed method are better. This is mainly due to the high resolution and receptive field (RF) of SPP-YOLO-v4, and the addition of SPP module in the connection layer retains the advantages brought by SPP. In terms of training time, SPP-YOLO-v4 is only slightly more than GMM. The CNN needs to train a lot of convolution operations, so its training time is longer.

Finally, the experiment uses data of 12 different audio formats to test and compare the four methods.  Table 4 provides the values of test accuracy and test time. It can be seen that the average accuracy of the method proposed in this study is 99.0%, and the average detection time is 0.449ss. Therefore, the proposed method achieves better performance among the four methods compared. It can be concluded that the upsampling and maximum pooling of SPP-YOLO-v4 brought significant benefits. Maximum pooling selects the maximum value from adjacent areas to slightly delete some maximum frequency noise in the audio sequence. Therefore, convolution subsampling can be better operated in the subsequent sampling layer. Through these advantages, SPP can improve the performance of the backbone network.

## 7. Conclusions

This study presents an audio recognition system suitable for music teaching environment. Use SPP to improve YOLO-v4 network architecture, that is to say, use SPP to select local areas on different scales of the same convolution layer to learn the characteristics of the multiscale system. In addition, the stacking method in ensemble learning is used to fuse independent submodels of two different channels. The experimental results show that the proposed method can improve the recognition accuracy of audio types and has better performance for different audio file formats. Due to the limitation of audio recording conditions, there are few audio types in the experimental dataset and the classification performance of audio files recorded by different devices has yet to be verified. More tests will be conducted on these two issues in the future.

## Figures and Tables

**Table 1:** The system settings.

Settings	Parameter
Audio channel	Single channel
Audio type	MFCC
Audio window length	40 ms
Audio frame shift	20 ms
Feature vector	Static MFCC + first-order + second-order
Feature vector length	60

**Table 2:** Generalization ability analysis of SPP-YOLO-v4.

Model	Audio file format	Accuracy				
		1	2	3	4	Average
YOLO-v4	MPEG	0.931	0.914	0.901	0.933	0.919
	MP3	0.889	0.961	0.894	0.880	0.906
	WMA	0.921	0.910	0.900	0.913	0.911

SPP-YOLO-v4	MPEG	0.951	0.969	0.961	0.959	0.956
	MP3	0.889	0.982	0.911	0.889	0.918
	WMA	0.937	0.989	0.919	0.938	0.945

**Table 3:** Performance comparison of different methods for different types.

Model	Loss value	Training time	Type	mAP (%)	TP	FP	Precision	Recall	*F*1
CNN	0.0143	2 h 40 min	*D*1	97.5	77	0	0.98	0.97	0.98
			*D*2	98.81	83	0			
			*D*3	99.92	62	1			
			*D*4	98.74	76	2			

GMM	0.0151	2 h	*D*1	97.53	78	0	0.98	0.96	0.97
			*D*2	100	83	0			
			*D*3	99.85	61	3			
			*D*4	98.01	75	3			

R-CNN	0.0131	2 h 20 min	*D*1	97.50	77	0	0.98	0.97	0.97
			*D*2	98.81	83	0			
			*D*3	99.92	59	0			
			*D*4	97.75	72	5			

SPP-YOLO-v4	0.0122	2 h 10 min	*D*1	97.51	78	0	0.99	0.99	0.99
			*D*2	98.82	83	0			
			*D*3	99.90	62	1			
			*D*4	98.94	79	3			

**Table 4:** Accuracy and detection time of different methods.

Format	CNN		GMM		R-CNN		SPP-YOLO-v4	
	Accuracy	Time (s)	Accuracy	Time (s)	Accuracy	Time (s)	Accuracy	Time (s)
CD	0.960	0.459	0.987	0.448	0.973	0.456	0.994	0.454
WAVE	0.791	0.453	0.963	0.442	0.826	0.459	0.993	0.452
AIFF	0.991	0.459	0.991	0.435	0.994	0.448	1.000	0.451
MPEG	0.970	0.473	0.990	0.448	0.994	0.457	0.997	0.443
MP3	0.951	0.445	0.990	0.447	0.931	0.452	0.982	0.457
MPEG-4	0.900	0.462	0.922	0.448	0.963	0.443	0.981	0.439
MIDI	0.907	0.460	0.870	0.449	0.901	0.451	0.982	0.448
WMA	0.787	0.453	0.880	0.462	0.841	0.460	0.996	0.449
RealAudio	0.869	0.457	0.982	0.464	0.947	0.447	0.993	0.433
VQF	0.863	0.447	0.961	0.442	0.866	0.459	0.992	0.450
AMR	0.957	0.453	0.960	0.443	0.990	0.459	0.991	0.451
AAC	0.881	0.452	0.632	0.471	0.961	0.466	0.989	0.459
Average	0.902	0.456	0.927	0.450	0.933	0.491	0.990	0.449

## Data Availability

The data used to support the findings of this study are available from the corresponding author upon request.
